# Accelerometer-measured physical activity and its impact on sleep quality in patients suffering from restless legs syndrome

**DOI:** 10.1186/s12883-021-02115-w

**Published:** 2021-02-26

**Authors:** A. K. Reimers, V. Heidenreich, H-J Bittermann, G. Knapp, C. D. Reimers

**Affiliations:** 1 Department of Sport Science and Sport, Friedrich-Alexander-University Erlangen-Nuremberg, Gebbertstraße 123b, 91058 Erlangen, Germany; 2 Practice for Neurology, Damm 49, 25421 Pinneberg, Germany; 3 Practice for Neurology, Harksheider Str. 3, 22399 Hamburg, Germany; 4 Department of Statistics, TU Dortmund University, Vogelpothsweg 87, 44227 Dortmund, Germany; 5 Practice for Neurology, Paracelsus-Klinik, In der Vahr 65, 28329 Bremen, Germany

**Keywords:** Exercise, Sports, Neurology, Actigraph, Germany, Sleep

## Abstract

**Background:**

The primary symptoms of restless legs syndrome (RLS) are sleep onset insomnia and difficulty to maintain sleep. Previous studies have shown that regular physical activity can reduce the risk of developing RLS. However, the relationship between physical activity and sleep quality parameters in individuals suffering from RLS has not yet been investigated by applying accelerometry. Thus, the present study investigates the impact of physical activity (measuring both intensity levels and duration of physical activity) during the day (7–12 h, 12–18 h, 18–23 h) on sleep quality in patients suffering from idiopathic RLS by applying a real-time approach.

**Methods:**

In a sample of 47 participants suffering from idiopathic RLS, physical activity and sleep quality were measured over one week using accelerometers. For data analysis, physical activity levels and step counts during three periods of the day (morning, afternoon, evening) were correlated with sleep quality parameters of the subsequent night.

**Results:**

This observational study revealed that in most instances physical activity was not correlated with sleep parameters (two exceptions exist: steps taken in the morning were negatively correlated with periodic leg movements during sleep, and physical activity in the evening was negatively correlated with total sleep time). The physical activity levels of the participants in this study, however, were unexpectedly high compared to population-level data and variance in physical activity was low. The average activity was 13,817 (SD = 4086) steps and 347 (SD = 117) minutes of moderate physical activity per day in females, and 10,636 (SD = 3748) steps and 269 (SD = 69) minutes of moderate physical activity in males, respectively. Participants did not engage in any vigorous physical activity.

**Conclusions:**

Further interventional studies are needed to investigate the daily effects of different intensities of physical activity on RLS symptoms.

## Background

The restless legs syndrome (RLS) is a common neurological long-term disease with a prevalence of 2.4 to 10.8% in adults [1]. The main characteristic of RLS is the longing to move one’s legs, caused by unpleasant sensations in the legs which improve slightly by moving them [2].

This disease develops either idiopathically, without a known cause, or symptomatically triggered by another condition (e.g., due to diabetic or uremic polyneuropathy in Parkinson’s and spinal cord diseases). In particular, RLS symptoms of unpleasant feelings and/or pain in the legs occur mainly in the evening or at night during physical rest. Common complaints are difficulty falling asleep and maintaining sleep [3]. Furthermore, periodic leg movements during sleep (PLMS) are the most important objective symptom of RLS [4, 5]. Most RLS patients experience PLMS and periodic leg movements during relaxed wakefulness which are characterized by repetitive involuntary movements of the legs [5–7]. PLMS are significantly correlated with short lasting awakenings [8]. The pathogenesis of RLS and the role of PLMS are not fully understood until now.

Moving the legs or walking around for a short time can help alleviate an individual’s discomfort [9]. Three prospective cohort studies [10–12] revealed that regular physical activity reduces the risk of developing RLS. Additionally, for patients suffering hemodialysis, exercise training has been shown to ameliorate symptoms of RLS [13–18]. DeMello et al. [19, 20] found that nighttime RLS symptoms in paraplegic individuals were significantly reduced after exercising twelve hours before sleeping. Contrastingly, Daniele et al. [21] found no correlation between physical activity and RLS complaints in diabetic individuals. In a randomized controlled trial of subjects with idiopathic RLS, Aukerman et al. [22] found significant relief of RLS complaints after strength and endurance training (administered twice a week over a period of twelve weeks). On the other hand, in another randomized trial, Harrison et al. [23] found no significant effects on RLS symptoms of a so-called tension and trauma release exercise program, conducted once a week over a period of six weeks (*n* = 9 participants in each group). The work of Franco et al. [24] suggests that physical exercise is a favorable non-pharmacological treatment for sleep-related movements disorders including RLS; regarding the potential mechanisms involved, the authors presume that exercise causes the release of beta-endorphins, which interact with opioid receptors in the brain, and dopamine, which leads to neurochemical balance in the brain. Although these processes may prevent the occurrence of PLMS, the mechanisms involved are not yet fully understood. The study by Franco et al. [24] also reports that increased levels of dopamine, following physical activity, have a similar effect on the body as dopaminergic agonists, medications that are commonly used in the pharmacological treatment of PLMS.

There is a variety of mechanisms by which exercise can positively effect sleep quality. These include both chronic and acute effects of exercise [25]. Acute effects of exercise on sleep quality, occurring after acute exercise, include the following: fatigue of the central nervous system, elevated body temperatures, heart rate changes, and heart rate variability changes [25]. Among individuals suffering from sleep-related movement disorders, the following effects were indicated after exercise: an increase of non-REM-sleep and shorter periods of REM-sleep, improvements in total sleep time, sleep efficiency, and number of sleep awakenings, and a decrease in PLMS symptoms [24]. Still, the question remains whether individuals with idiopathic RLS also experience improvements in sleep quality and night-time PLMS symptoms, following physical activity during the proceeding day.

This potential relationship is hypothesized to occur on a short-term basis, within a 24-h period [26]; physical activity during a day is assumed to affect sleep quality during the following night. In previous studies with healthy individuals [26] and individuals suffering from RLS [14, 17], physical activity and sleep quality were often averaged over multiple days. This method overlooks day-to-day variations in physical activity which are necessary to determine whether physical activity influences sleep quality on a micro-scale level (within 24 h) [27]. Thus, to investigate this relationship, day-to-day within-person variations should be considered and physical activity and sleep quality parameters need to be observed on a daily basis.

Accelerometers are small portable devices, consisting of electronic sensors which measure acceleration (the rate at which the velocity of an object changes over time). The use of accelerometers in physical activity research has increased significantly since the mid-1990s [28]. They are especially helpful in examining day-to-day variations in physical activity [29]. The advantage of accelerometry is that dense data can be collected over a period of multiple days. This allows for a detailed examination of daily behavior and its variations over the period of one day or several days. Although laboratory-based polysomnography is considered to be the gold standard for diagnosing sleep parameters, accelerometers can also be used to capture parameters of sleep quality, such as PLMS [30]. Regarding sleep quality measurements, accelerometers can be placed at the ankle or arches of both feet to detect limb movements during sleep. The advantages of accelerometry over polysomnography include the possibility to record sleep parameters over periods of more than 24 h without the need to replace the sensors or batteries [31]. Furthermore, an individual’s sleep quality can be determined while sleeping in their natural home environment [7]. The accuracy of accelerometry, however, is restricted in comparison to polysomnography [31].

Accelerometers have previously been applied in studies with patients suffering from RLS [32, 33]. Nevertheless, the research in this area is limited. Studies which use accelerometers to capture physical activity in RLS patients are even harder to come by [34]. Nonetheless, research has established that accelerometers are valid tools to capture physical activity, for example in persons with multiple sclerosis [35] or Parkinson’s disease [36].

The present observational study aimed to investigate the relationship between daytime physical activity, insomnia, and other sleep parameters in individuals suffering from idiopathic RLS by applying a real-time approach and considering day-to-day variations in physical activity. One the one hand, it aimed to analyze the effects of previous day physical activity, during different times of day, on sleep quality. On the other hand, the effects of different intensities and levels of previous day physical activity on sleep quality were analyzed. Finally, the overall levels of physical activity in subjects with idiopathic RLS were analyzed and compared with epidemiological data from the German population.

## Methods

### Study design and recruitment

As physical activity varies substantially over the course of a week [37, 38], an observational study design was considered appropriate to investigate the daily relationship of physical activity and sleep quality in individuals suffering from RLS. Both physical activity levels and sleep quality of a given individual vary over the course of a week [39]. Thus, the co-variation between physical activity during the day and quality of sleep during the subsequent night should be taken into account. Accordingly, an observational study based on a sample of patients suffering from idiopathic RLS was conducted. Data collection took place between August 2017 and December 2018. The participants were recruited from three neurological practices in northern Germany (Bremen, Hamburg, and Pinneberg). Inclusion criteria were an age of at least 18 years, the provision of informed consent, and an established diagnosis of idiopathic RLS. We used the Restless Legs Syndrome Diagnosis Index (RLS-DI), with a cut-off of ≥11, as a diagnostic tool for testing the eligibility of the participants. Exclusion criteria were pregnancy, symptomatic RLS, including metabolic abnormalities, e.g., renal insufficiency, diabetes mellitus, iron deficiency (ferritin concentration < 50 μg/l), as well as all other diseases that limit physical activities during daily life. Drugs which can potentially aggravate RLS symptoms, such as antidepressants (e. g., citalopram, paroxetine), or benefit them, such as sedatives (e. g., benzodiazepines), were not taken by the participants. No preselection of the participants regarding their physical activity was made. Physical activity levels of participants were unknown at the time they were included in the examination.

The study was approved by the ethic committees of Georg-August-University Göttingen (no. 17/2/2016) and the Medical Association of Thuringia (59,838/2017/142), and was conducted according to the Declaration of Helsinki. All study participants provided written informed consent to participate and were informed in detail about the study and data management by the neurologist of the practices where they were recruited.

### Data collection

Data collection took part over a period of eight days. Initially, participants were instructed and informed about the study aims and data collection procedure by the neurologists in the neurological practices they regularly attend. Participants were informed that this study aims to identify relationships between daytime physical activity and sleep quality. Additionally, participants were asked not to change their medication or habitual lifestyle throughout the data collection period. Due to the risk of potentially intensifying RLS complaints, asking participants to stop taking their RLS medication was deemed unethical.

While discussing informed consent, participants received three ActiGraph Link accelerometers (ActiGraph Corporation, Pensacola, FL, USA). The ActiGraph Link is a small, commercially available, tri-axial, wearable inertial measurement unit. ActiGraph devices are frequently used and validated in both capturing physical activity [40] and measuring sleep parameters in free-living conditions [30, 41, 42]. As a result, these devices were regarded as useful for this study. One of the accelerometers, which was provided to capture physical activity levels, was equipped with a wristband. The other two accelerometers, that were provided to objectively assess sleep parameters during the night, were not attached to either a belt or band. The participants were instructed as to how and when to wear these accelerometers during the following eight consecutive days and nights. Furthermore, they were instructed to document their sleep quality during the eight day data collection period (one the first day the accelerometers were handed out and one the eighth day they were returned). Participation was voluntary and anonymous, and participants were informed about data security regulations prior to the data collection and analysis. Participants were instructed verbally and in written form how to use the accelerometers during the data collection period.

### Measures

#### Objectively measured actual physical activity

Assessments of physical activity were obtained using an accelerometer that was worn on the wrist of the participants’ dominant hand. A 1 s-interval was used to capture “activity counts” of the respective participant. Activity counts were converted to average minutes spent resting, or in light (< 3 metabolic equivalents (METs)), moderate (3–6 METs), vigorous (6–9 METs), and very vigorous (> 9 METs) physical activity. Cut-offs for different intensity levels of physical activity were set according to the recommendations of Sasaki et al. [43]: light activity 0–2690 counts per minute (cpm), moderate activity 2691–6166 cpm, intensive activity 6167–9642 cpm, and very intensive activity ≥9643 cpm. The minutes spent in each level were calculated for three separate time periods over each day (morning: 6 AM − 12 noon., afternoon: 12 noon – 6 PM, and evening: 6 PM – 11 PM). For each time period a variable was built which accounted for total minutes of light, moderate, and vigorous physical activity. Additionally, the number of steps taken during each of the three time periods of the day were counted, and total daily steps were calculated.

#### Objectively measured sleep quality

In order to assess PLMS, according to the manufacturer’s description [Actilife 6 User’s Manual], two accelerometers were placed at the arches of the feet before sleep, secured with medical tape; both the medical tape and the accelerometers were provided by the neurologists. For assessment of sleep quality, an additional accelerometer was worn on the wristband. The parameters that were recorded were the number of sleep interruptions (accelerometer at the wrist), PLMS per hour, total sleep time, and sleep efficacy (duration of sleep divided by the duration of time spent lying in bed) (accelerometer at the arches of the feet).

### Statistical analyses

An independent t-test was performed to identify significant between group differences (between males and females). The statistical evaluation did not include the first and last day of the data collection period, as recording of these both days did not cover a whole day. In total, six days were considered for statistical analyses. For each participant, the mean and median values and corresponding measures of dispersion of the steps taken were calculated. The duration, in minutes per day, of each physical activity intensity category (light, moderate, intensive, and very intensive) was also calculated.

Considering each day of the data collection period (not the total investigational period), the associations (Pearson-Bravais correlation coefficients) between the number of steps or minutes of physical activity, respectively, and the sleep parameters were analyzed. Afterwards, the correlation coefficients were z-transformed. Due to the fact that not all participants documented six complete investigational days, the z values were weighted by the number of days for which data was available. Accordingly, the mean values of the z values of all participants were calculated and re-transformed into correlation coefficients.

Two-by-two-table tests were performed by using Fisher’s exact test. The correlation coefficients between school education, mean daily steps, and moderate physical activity, were calculated using Spearman’s rank correlation coefficient. A level of 0.05 was set as a threshold to determine statistical significance.

## Results

### Descriptive results

Sixty-two patients suffering from RLS initially expressed interest in participating in the study. Five of them withdrew their interest during the discussion with their neurologists, before inclusion and exclusion criteria was considered. Of the remaining 57 potential study participants (40 women and 17 men) six women and four men had to be excluded due to the following reasons: indications of a secondary RLS, such as iron deficiency, vitamin B12 deficiency, or diabetes mellitus; lack of attendance when the accelerometers were handed out; abandonment of study, e.g., due to another acute disease; and provision of invalid accelerometric data. Consequently, usable data was delivered by 34 female and 13 male participants (*N* = 47). The mean age of the participants was 65.4 ± 10.0 years in women and 55.3 ± 14.7 years in men. Descriptive data of the participants is presented in Table 1. With one exception, each participant received medication during the data collection period (in order to alleviate RLS symptoms) and all participants confirmed not having changed their treatment throughout the data collection period.

**Table 1:** Biometrical, behavioral, and sleep data of all participants

	Age [years]	Disease duration [years]	BMI[kg/m^**2**^]	Steps (8 AM– 11PM)	Steps: Individual variance^a^ [%]	Moderate physical activity (8 AM–11 PM) [min.]	Moderate physical activity: Individual variance^a^ [%]	Number of interruptions of sleep	Total sleep time [h]	Sleep efficacy [%]	PLMS / h
**Women (*N*** **= 34)**											
M (SD)	65.4 (10.0)	11.8 (12.0)	26.9 (4.8)	13,817 (4086)	15 (7)	347 (117)	18 (9)	17.8 (7.2)	5.9 (2.0)	81.2 (15.7)	7.6 (12.1)
Median	64.6	8	25.7	13,370	14	332	15	17.0	5.7	81.0	2.9
1st and 3rd quartile	56.3–74.8	3–15	23.5–29.8	10,565 – 16,794	11–19	257–419	12–21	12.3–22.0	4.6–7.2	73.7–86.8	1,6 – 6,7
**Men (*N*** **= 13)**											
M (SD)	55.3 (14.7)	15.8 (15.6)	28.5 (4.9)	10,636 (3748)	28 (15)	269 (96)	25 (6)	16.1 (7.5)	6.2 (2.0)	79.4 (11.2)	5.7 (7.9)
Median	55.3	10	29.9	9924	24	245	25	17.0	5.7	81.5	3.0
1st. and 3rd quartile	40.3–66.7	5–19	24.3–31.3	8034 – 12,843	18–30	197–321	21–30	12.0–22.0	5.0–6.9	72.9–88.0	1.0–7.0

*Note*: h = hours; M = mean value; min = minutes; N = sample size; *PLMS* periodic leg movements, *SD* standard deviation; ^a^ individual variance = intra-individual variance of physical activity from day to day

Female participants were significantly older than male participants (*p* = 0.035). Nine participants only completed compulsory schooling (basic secondary school), 13 females and two males completed secondary school, two females successfully passed the high school, and ten female and two male participants obtained a university degree. Nineteen participants (40%) were employed, and 28 participants were pensioners (60%).

### Level of physical activity

None of the participants performed vigorous or very vigorous physical activity (accelerometer cut-offs > 6166 cpm). The number of daily steps significantly correlated with the duration of moderate physical activity (r = 0.88, *p* < 0.001). The participants´ body mass indexes neither correlated significantly with daily steps (women: r = − 0.28, men: r = − 0.05) nor with moderate physical activity (women: r = 0.02; men: r = 0.30). Both the mean number of weekly footsteps (*p* = 0.003) and the mean duration of weekly moderate activity (*p* = 0.012) were significantly higher in female participants, compared to their male counterparts. The age of participants did not correlate with the mean daily steps (women: r = − 0.11, men: r = 0.07) or the mean duration of moderate physical activity (women: r = − 0.03, men: r = − 0.45). The mean number of steps also did not correlate with education (women: r = 0.27, men: r = 0.12).

### Sleep quality

The mean total sleep time, which was assessed objectively, was 361 min (SD = 10 min; median value: 342 min; 1st and 3rd quartile: 284 and 430 min, resp.). Complete accelerometer sleep data was available from 40 participants regarding PLMS, and 45 participants regarding awakenings, total sleep time, and sleep efficacy.

### Analytical statistics

#### Impact of physical activity on RLS symptoms

From a subjective perspective, twenty-five participants (17 women, 8 men) reported that their physical activity had no impact on their RLS complaints, 13 participants (11 women, 2 men) were not sure regarding the possible impact, 8 participants (5 women, 3 men) reported that physical activity helped to alleviate their complaints, and one female participant reported physical activity negatively impacting her RLS complaints.

The statistical data analyses revealed low, but significant, negative correlation coefficients between the number of steps, the amount of moderate physical activity in the evening, and the total sleep time (more steps resulted in a shorter sleep time). Significant, negative correlation coefficients were also found between the number of steps in the morning and nighttime PLMS (the more steps taken in the morning, the less PLMS). No further significant correlations were found between daily step and moderate physical activity during any of the three observational periods (6 AM – 12 noon, 12 noon – 6 PM, 6 PM – 11 PM). Furthermore, no additional significant correlations were found among the physical activity levels and any of the objectively measured sleep parameters (total sleep time, number of interruptions, sleep efficacy, number of PLMS) (Table 2 and Table 3).

**Table 2:** Correlation between steps and sleep quality parameters

		Number of interruptions of sleep			Total sleep time			Sleep efficacy			PLMS / h		
		N	R	p	N	R	p	N	R	P	N	R	p
Steps 6 AM - 12 noon	All	45	− 0.0040 (95% CI: − 0.1809 – 0.1731)	0.9648	45	− 0.1034 (95% CI: − 0.2754 – 0.0750)	0.2555	45	− 0.0898 (95% CI: − 0.2626 – 0.0887)	0.3241	**40**	**− 0.1958 (95% CI: − 0.3768 – − 0.0003)**	**0.0496**
	Males	12	− 0.1684 (95% CI: − 0.4933 – 0.1977)	0.3682	12	− 0.0915 (95% CI: − 0.4318 – 0.2717)	0.6274	12	− 0.1378 (95% CI: − 0.4692 – 0.2277)	0.4630	8	− 0.1241 (95% CI: − 0.5102 – 0.3036)	0.5768
	Females	33	0.0465 (95% CI: − 0.1566 – 0.2457)	0.6556	33	− 0.1071 (95% CI: − 0.3021 – 0.0966)	0.30266	33	− 0.0751 (95% CI: − 0.2725 – 0.1284)	0.4707	32	− 0.2138 (95% CI: − 0.4129 – 0.0048)	0.0551
Steps 12 noon - 6 PM	All	44	− 0.0928 (95% CI: − 0.2676 – 0.0879)	0.3140	44	0.0550 (95% CI: − 0.1255 – 0.2319)	0.5518	44	0.0593 (95% CI: − 0.1212 – 0.2361)	0.5205	39	0.0004 (95% CI: − 0.1970 – 0.1978)	0.9967
	Males	11	−0.3259 (95% CI: − 0.6185 – 0.0462)	0.0846	11	0.0602 (95% CI: − 0.3133 – 0.4175)	0.7587	11	−0.0854 (95% CI: − 0.4382 – 0.2902)	0.6625	7	− 0.1601 (95% CI: − 0.5449 – 0.2805)	0.4815
	Females	33	−0.0231 (95% CI: − 0.2246 – 0.1804)	0.8258	33	0.0535 (95% CI: −0.1508 – 0.2533)	0.6097	33	0.1005 (95% CI: −0.1043 – 0.2971)	0.3361	32	0.0403 (95% CI: −0.1810 – 0.2578)	0.7232
Steps 6 PM - 11 PM	All	45	−0.1116 (95% CI: − 0.2817 – 0.0653)	0.2158	**45**	**− 0.2221 (95% CI: − 0.3827 – − 0.0483)**	**0.0126**	45	− 0.0876 (95% CI: − 0.2592 – 0.0894)	0.3322	40	− 0.1081 (95% CI: − 0.2905 – 0.0945)	0.3094
	Males	12	0.0386 (95% CI: − 0.3088 – 0.3769)	0.8326	12	− 0.1577 (95% CI: − 0.4753 – 0.1963)	0.3838	12	−0.2096 (95% CI: − 0.5158 – 0.1441)	0.2439	8	− 0.2120 (95% CI: − 0.5669 – 0.2093)	0.3239
	Females	33	−0.1599 (95% CI: − 0.3501 – 0.0431)	0.1221	**33**	**−0.2427 (95% CI: − 0.4235 – − 0.0432)**	**0.0176**	33	− 0.0470 (95% CI: − 0.2462 – 0.1560)	0.6518	32	− 0.0716 (95% CI: − 0.2855 – 0.1491)	0.5265
Steps 6 AM - 11 PM	All	44	− 0.1411 (95% CI: − 0.3124 – 0.0391)	0.1245	44	− 0.0659 (95% CI: − 0.2423 – 0.1147)	0.4752	44	− 0.0284 (95% CI: − 0.2066 – 0.1516)	0.7587	39	− 0.1516 (95% CI: − 0.3389 – 0.0472)	0.1343
	Males	11	− 0.0322 (95% CI: − 0.6156 – 0.0509)	0.0891	11	0.0299 (95% CI: − 0.3403 – 0.3921)	0.8787	11	−0.1097 (95% CI: − 0.4578 – 0.2676)	0.5745	7	− 0.1989 (95% CI: − 0.5725 – 0.2431)	0.3795
	Females	33	−0.0871 (95% CI: − 0.2847 – 0.1176)	0.4048	33	− 0.0932 (95% CI: − 0.2903 – 0.1116)	0.3728	33	− 0.0051 (95% CI: − 0.2075 – 0.1978)	0.9615	32	− 0.1398 (95% CI: − 0.3489 – 0.0824)	0.2168

Note: significant correlations are shown in bold

**Table 3:** Correlation between moderate physical activity and sleep quality parameters

		Number of interruptions of sleep			Total sleep time			Sleep efficacy			PLMS / h		
		N	R	p	N	R	p	N	R	p	N	R	p
MPA 6 AM - 12 noon	All	45	− 0.0040 (95% CI: − 0.1809 – 0.1731)	0.9648	45	− 0.0977 (95% CI: − 0.2701 – 0.0807)	0.2828	45	− 0.0207 (95% CI: − 0.1970 – 0.1569)	0.8207	40	− 0.0917 (95% CI: − 0.2821 – 0.1056)	0.3625
	Males	12	−0.1684 (95% CI: − 0.4933 – 0.1977)	0.3682	12	−0.1531 (95% CI: − 0.4813 – 0.2128)	0.4142	12	− 0.1600 (95% CI: − 0.4867 – 0.2061)	0.3932	8	−0.0108 (95% CI: − 0.4212 – 0.4032)	0.9613
	Females	33	0.0465 (95% CI: −0.1566 – 0.2457)	0.6556	33	−0.0808 (95% CI: − 0.2778 – 0.1228)	0.4376	33	0.0221 (95% CI: −0.1802 – 0.2227)	0.8320	32	−0.1123 (95% CI: − 0.3228 – 0.1087)	0.3191
MPA 12 noon - 6 PM	All	44	−0.0928 (95% CI: − 0.2676 – 0.0879)	0.3140	45	−0.0146 (95% CI: − 0.1912 – 0.1628)	0.8728	45	0.0039 (95% CI: − 0.1733 – 0.1808)	0.9663	40	0.0832 (95% CI: −0.1141 – 0.2742)	0.4090
	Males	11	−0.3259 (95% CI: − 0.6185 – 0.0462)	0.0846	12	−0.0891 (95% CI: − 0.4246 – 0.2679)	0.6303	12	− 0.1318 (95% CI: − 0.4594 – 0.2274)	0.4753	8	−0.1671 (95% CI: − 0.5345 – 0.2534)	0.4395
	Females	33	−0.0231 (95% CI: − 0.2246 – 0.1804)	0.8258	33	0.0092 (95% CI: −0.1938 – 0.2114)	0.9300	33	0.0473 (95% CI: −0.1568 – 0.2475)	0.6517	32	0.1510 (95% CI: −0.0711 – 0.3588)	0.1818
MPA 6 PM - 11 PM	All	45	−0.1116 (95% CI: − 0.2817 – 0.0653)	0.2158	**45**	**−0.2485 (95% CI: − 0.4064 – − 0.0762)**	**0.0051**	45	−0.1305 (95% CI: − 0.2993 – 0.0461)	0.1471	40	− 0.0679 (95% CI: − 0.2589 – 0.1283)	0.4988
	Males	12	0.0386 (95% CI: −0.3088 – 0.3769)	0.8326	12	−0.2741 (95% CI: − 0.5643 – 0.0764)	0.1234	12	−0.2834 (95% CI: − 0.5711 – 0.0664)	0.1106	8	− 0.1786 (95% CI: − 0.5429 – 0.2422)	0.4080
	Females	33	−0.1599 (95% CI: − 0.3501 – 0.0431)	0.1221	**33**	**−0.2401 (95% CI: − 0.4213 – − 0.0405)**	**0.0188**	33	−0.0789 (95% CI: − 0.2761 – 0.1246)	0.4481	32	− 0.0377 (95% CI: − 0.2539 – 0.1822)	0.7393
MPA 6 AM - 11 PM	All	44	− 0.1411 (95% CI: − 0.3124 – 0.0391)	0.1245	45	−0.0805 (95% CI: − 0.2553 – 0.0995)	0.3811	45	− 0.0335 (95% CI: − 0.2107 – 0.1459)	0.7160	40	0.0164 (95% CI: − 0.1806 – 0.2121)	0.8718
	Males	11	−0.0322 (95% CI: − 0.6156 – 0.0509)	0.0891	12	−0.1255 (95% CI: − 0.4647 – 0.2459)	0.5122	12	− 0.1397 (95% CI: − 0.4760 – 0.2323)	0.4650	8	−0.0858 (95% CI: − 0.4810 – 0.3384)	0.7006
	Females	33	−0.0871 (95% CI: − 0.2847 – 0.1176)	0.4048	33	−0.0670 (95% CI: − 0.2660 – 0.1375)	0.5220	33	− 0.0017 (95% CI: − 0.2043 – 0.2010)	0.9870	32	0.0429 (95% CI: − 0.1785 – 0.2602)	0.7062

*Note*: MPA = moderate physical activity; significant correlations are shown in bold

## Discussion

In the present study, the physical activity of subjects suffering from idiopathic RLS and its impact on RLS-related sleep quality was analyzed over one week, by considering within-day variations. To this end, participants wore accelerometers to capture their daily steps and physical activity (during three periods of the day) and to capture their sleep quality (throughout the night over a time span of one week).

This study did not ascertain statistical correlations between the number of daily steps, moderate physical activity at various times of the day, and any parameter of sleep quality. Two exceptions exist: a greater number of steps taken in the morning was correlated with less PLMS and increased physical activity in the evening was correlated with a shortened total sleep time). Overall, the present study does not reveal an obvious correlation between day-time physical activity and sleep quality in idiopathic RLS.

However, it is notable that the levels of moderate physical activity were extremely high in the study population compared to epidemiological data from similar age groups; they varied only slightly, both between participants (inter-individual) and also individually between different observation days (intra-individual). In the current study, the mean value for daily steps was around 14,000 steps in females and around 10,600 steps in males, respectively. In comparison, worldwide data comprising of 68 days and over 700,000 people [44] showed that the mean number of daily steps, in each of 111 nations included, was around 4000 to 6000 steps – except for Hongkong where the population walked around 7000 steps per day on average. Regarding the recommended level of steps per day [45], daily steps of 10,000 to 12,499 are considered as active and above 12,500 as highly active.

The World Health Organization [46] recommends that adults participate in at least 150 min of moderate physical activity per week. Among women in Germany, nationwide representative data revealed that only 15.5% are physically active at least 150 min per week, whereas the corresponding percentage in men is 25.4% [47].

Conversely, physical activity levels among female participants in our study considerably exceeded the recommended levels of physical activity; the majority of the participants walked more the 10,000 steps per day, which is considered as the benchmark for an active lifestyle [45, 48]. On average, female participants reached more than twice, and males 1.5 times the amount of recommended levels of physical activity. Similarly, the average number of daily steps among all participants was approximately twice as high as the general population when compared to epidemiological data from previous studies [44, 49]. Furthermore, female participants in the current study were more active and accumulated more daily steps than the males. This may be due to the fact that they were on average around ten years older than the males, and therefore more often unemployed and potentially had more time to invest in physical activity. However, other studies monitoring the physical activity of general populations in Germany, and worldwide, have consistently revealed that levels of physical activity or step counts decrease with increasing age, in people from the age of 50–60 [47, 49, 50]. Thus, the higher levels of physical activity in the female participants (with older average ages) compared to the males seems to be an anomaly of this study population. Although we do not have any evidence to explain the high levels of moderate physical activity among our study population, we assume that the study participants have experienced the benefits of moderate physical activity, and as a result, have developed daily routines with high levels of daily moderate physical activity. The positive experience and beneficial effects of moderate physical activity reported by the participants is in line with the results of studies – which show a positive impact of physical activity on RLS symptoms [9, 19, 20, 51].

Although their levels of moderate physical activity were remarkably high, none of the participants in the current study performed vigorous physical activity. One possible explanation could be that participants willingly or unconsciously avoided highly intensive physical activity, because they may expect it to cause negative consequences with regards to their RLS symptoms. However, previous studies of non-RLS patients, have concluded that high-intensity exercise (even when performed in the evening) does not affect sleep [52, 53]. Another explanation for the lack of vigorous physical activity among our study population is the use of a high cut-off point to define vigorous physical activity. The cut-off point of 6166 cpm, which was applied in this study, is based on existing evidence [43]. Other studies, however, have suggested lower cut-offs points (6000 cpm or 5725, respectively) [54, 55]. Particularly among older age groups, no consensus has been made on the most appropriate cut-off points for the classification of intensity levels of physical activity [56].

### Strength and limitations

This study has some strengths that should be considered: To begin with, to the best of our knowledge, this is the first study investigating the relationship between physical activity and sleep quality in patients suffering from RLS by applying a real-time approach and objective measures. By capturing levels of physical activity and sleep quality in real time and considering day-to-day variations, short-term effects, which have not been previously investigated, could be analyzed. Secondly, this study is based on an objective measurement of physical activity; both physical activity and sleep were simultaneously measured using accelerometry, which has been shown to be a valuable method for this purpose [41]. Although measures on sleep quality may be less accurate then polysomnography, which is often considered a gold standard [33, 57], accelerometry is a cost-efficient alternative to polysomnography for monitoring sleep disturbances [58] and PLMS [33, 57, 59, 60]. Additionally, accelerometers can be used to capture sleep quality parameters in a real-life home environment. Third, different times of the day have been taken into account when analyzing the relationships of physical activity and sleep quality.

Some limitations of our study should also be considered: Firstly, the impact of the medication taken by participants on the results could not be assessed or quantified. Secondly, participants in this study maintained similar patterns of physical activity during the data collection, with only small variations from day to day. Thus, the inter-individual and intra-individual variance of physical activity in the sample was low, which could preclude conclusions about the relationship between physical activity and sleep quality. Thirdly, we only tested for linear relationships between physical activity and sleep quality without taking other relationships into account, for example a U-shaped relationship. During personal contact with the participants, some of them reported experiencing such a relationship. Fourthly, the power of our study may be too low to revealing statistically significant results.

To investigate the effects of daily physical activity on RLS symptoms interventional studies with different intensities of physical activities, at different points of time during the day, are therefore needed.

## Conclusion

To the best of our knowledge, the present study is the first study to investigate the effects of physical activity on sleep quality in patients with idiopathic RLS and to report their physical activity levels using a real-time approach based on accelerometry. No association was found between physical activity and sleep quality. This could potentially be explained by the low variance in physical activity levels and step counts within the sample. It is worth noting participants in the study sample were extremely active (at a moderate intensity) compared with activity levels of the general population. Finally, in the event that further studies show a benefit of physical activity on RLS symptoms, this condition could be an potential model for patient self-treatment through regular physically active.

## Data Availability

The datasets used and/or analyzed during the current study not publicly available due to privacy reasons of patients, but are available from the corresponding author on reasonable request.
